# Variability in Oral Iron Prescription and the Effect on Spanish Mothers’ Health: A Prospective Longitudinal Study

**DOI:** 10.3390/jcm10215212

**Published:** 2021-11-08

**Authors:** Regina Ruiz de Viñaspre-Hernández, José Antonio García-Erce, Francisco José Rodríguez-Velasco, Vicente Gea-Caballero, Teresa Sufrate-Sorzano, María Elena Garrote-Cámara, Raquel Urra-Martínez, Raúl Juárez-Vela, Michał Czapla, Iván Santolalla-Arnedo

**Affiliations:** 1 Centro de Salud “Cascajos”, Servicio Riojano de Salud, Government of La Rioja, 26002 Logroño, Spain; reruizde@unirioja.es; 2 GRUPAC, Biomedical Research Center of La Rioja (CIBIR), Research Unit on Health System Sustainability (GISSOS), Department of Nursing, University of La Rioja, 26004 Logroño, Spain; teresa.sufrate@unirioja.es (T.S.-S.); maria-elena.garrote@unirioja.es (M.E.G.-C.); ivan.santolalla@unirioja.es (I.S.-A.); 3 Research Institute Idi-Paz, PBM Group, 28046 Madrid, Spain; ja.garcia.erce@navarra.es; 4 Hematologist, Bank of Blood and Tissue, Government of Navarra, 31015 Pamplona, Spain; 5 Department of Nursing, Faculty of Medicine, University of Extremadura, 06006 Badajoz, Spain; fcorodriguezv@unex.es; 6 Faculty of Health Sciences, International University of Valencia, 46010 Valencia, Spain; 7 Servicio Riojano de Salud, Hospital San Pedro, Government of La Rioja, 26006 Logroño, Spain; rurra@riojasalud.es; 8 Laboratory for Experimental Medicine and Innovative Technologies, Department of Emergency Medical Service, Wroclaw Medical University, 51-516 Wroclaw, Poland; michal.czapla@umw.edu.pl; 9 Institute of Heart Diseases, University Hospital, 50-566 Wroclaw, Poland

**Keywords:** prescription, medicine, iron, anemia, gynecology

## Abstract

Background: No consensus exists regarding the hemoglobin (Hb) values that define postpartum anemia. Knowledge is currently lacking regarding prescription and consumption practices, which prevents evaluating the rational use of iron supplementation postpartum. Aim: In this study, our objective was to describe this practice and analyze its association with maternal health outcomes. Methods: A prospective observational study was conducted with 1010 women aged between 18 and 50. The hemoglobin value on the first postpartum day; the prescription schedule at hospital discharge; iron consumption; and data on hemoglobin, serum ferritin, maternal fatigue, type of breastfeeding, and perceived health six weeks after delivery were collected. Findings: Oral iron was prescribed to 98.1% of mothers with anemia and 75.8% without anemia. At the same Hb value, the maximum amount of total iron prescribed was between 8 and 10 times greater than the minimum amount. Iron intake was significantly lower than prescribed (*p* < 0.01). At six weeks, anemic mothers who took iron presented a 3.6-, 3-, and 2.4-times lower probability of iron deficiency, anemia, and abandoning breastfeeding, respectively. Discussion: Postpartum iron intake shows a protective effect on iron deficiency and anemia at six weeks, but not on fatigue or self-perceived health level. Conclusion: We conclude that there is wide variability in the prescription regimen. Oral iron supplementation can benefit mothers with anemia and harm those without. Subsequent studies should further explore the Hb figure that better discriminates the need for postpartum iron.

## 1. Introduction

Anemia is a condition where the hemoglobin (Hb) levels are much lower than normal, which decreases the ability of the blood to deliver oxygen to tissue to meet physiological needs. In the postpartum period, anemia is the result of unresolved prenatal anemia and/or excessive blood loss that is associated with childbirth [1]. If the state of anemia persists after the postpartum period, it can deteriorate maternal health and make parenting difficult [2,3,4,5].

No consensus exists regarding the Hb values that define postpartum anemia. The clinical practice guidelines accept different cutoff points at the beginning of postpartum from Hb < 8, Hb < 10, Hb < 10.5, Hb < 11, or Hb ≤ 12 g/dL [6,7,8,9,10]. This discrepancy leads to differences in the postpartum iron supplementation criteria, with the Hb values between 10 and 12 g/dL being where the definition between pathological and physiological is disputed [11].

The populations observed, the cut-off value of Hb used, and the postpartum time in which Hb is measured make the prevalence data heterogeneous. The World Health Organization [12], taking an Hb value of <10 g/dL as a reference, estimates the prevalence of postpartum anemia to be between 10% and 30% in developed countries and above 50% in low- and middle-income countries.

Oral iron therapy is the first line of treatment in most cases. Studies have shown that consuming iron leads to higher levels of Hb in less time, but also that it can cause side effects in mothers. The rational use of iron supplements postpartum involves their appropriate use, so that their selection, dose, and duration are in accordance with the relevant guidelines; suitable for clinical purposes; at the lowest cost to the provider, community, and patient; and are correctly dispensed and administered properly [13]. Spain does not have its own guidelines, and the management of postpartum anemia varies between hospitals; hospitals routinely measure the Hb level one day after delivery to guide the prescription of iron. However, the practice of prescribing iron, the actual consumption of it in the postpartum period, and its clinical impact have not been described, as is also the case in countries where guidelines exist [14].

In this study, we aimed to describe the prescription and actual iron consumption in the postpartum period and analyze the association between iron consumption and iron deficiency, anemia, maintenance of breastfeeding, fatigue, and self-perceived health status in mothers starting postpartum with and without anemia.

## 2. Materials and Methods

We conducted a longitudinal descriptive study on the prescription of oral iron, actual consumption, and the effect of iron intake on maternal health.

### 2.1. Study Subjects

Women who had experienced childbirth in La Rioja, Spain, were included in our study. The sample size was calculated based on a population probability of the prevalence of postpartum anemia of 25% [15], a precision of 5%, a confidence level of 95%, and a percentage of loss of 20%. With these assumptions, the estimated minimum sample size was 275 women per year. The data were collected between October 2017 to February 2020 and from January to March 2021. A total of 1181 women were screened in the postnatal period. 112 mothers used any supplementation containing iron and 3 had intestinal malabsorption or bariatric surgery. These 115 (10.8%) women were excluded from the study.

All women who visited the midwife in the first week postpartum that agreed to participate in the study had a complete blood count and serum ferritin analysis at six weeks postpartum. They were also educated to understand the purposes of the study and provided informed consent. Four health centers, which were representative of the broader regional population due to their geographical location and the diversity of the population served, continuously and simultaneously collected the data.

Mothers who used any supplementation containing iron and mothers that were diagnosed with intestinal malabsorption or bariatric surgery were excluded from the study. The La Rioja Clinical Research Ethics Committee (CEICLAR) approved the study.

### 2.2. Data Collection

The data was collected, continuously and simultaneously, in four health centers in the region. These centers were selected because they serve a large and diverse population of women in the community due to their different locations in the community. All the women who attended the midwife’s office in the first week postpartum, who agreed to participate in the study and signed the informed consent, and who agreed to carry out an analysis six weeks after delivery were included in the study. Blood was subjected to a complete blood count and serum ferritin measurement. The only exclusion criteria were: having language or cognitive difficulties that prevented voluntary participation in the study and giving consent to it, being diagnosed with a malabsorption syndrome that prevented or discouraged the use of oral iron or having a surgical resection of part of the stomach or small intestine.

The data were collected during visits of the midwives, in the first and sexth postpartum weeks. In the first visit, the sociodemographic data of the women (age and place of residence), the clinical data (parity and type of delivery), the analytical data of the pregnancy, and the hospital postpartum (Hb and serum ferritin (SF)) levels, as well as the iron prescription, were collected. The report that was given to mothers at discharge detailed the postpartum oral iron prescription regimen (a daily dose of elemental iron in mg and treatment time in days). The daily dose was multiplied by the number of days to calculate the total prescribed iron. During this visit, the request to repeat the blood count and serum ferritin 40 days following delivery and the appointment for the next visit was submitted.

In the second visit, the actual iron consumption data and the analytical data (Hb and SF levels), as well as the clinical data (lactation, fatigue, and health), were collected. To quantify consumption, the number of doses consumed was calculated, the women provided the blisters, and they reported on their consumption practice: daily dose and the days of consumption.

Anemia in the first 24 h postpartum was defined with a Hb value < 10 g/dL. At 6 weeks after delivery, anemia was defined as Hb < 12 g/dL and iron deficiency was characterized by an SF value of < 15 µg/L. The mothers self-reported maintaining breastfeeding. The level of fatigue was measured with a questionnaire translated to and validated in Spanish [16], and the self-perceived general health was measured with a visual analog scale (VAS) of 0–100, which indicates the worst possible health status to the best possible health status [17].

### 2.3. Statistical Analysis

The variables were summarized using means and standard deviations (SD), the median and range, or frequencies and percentages, depending on the type of variable and the type of distribution. To measure the differences between women with and without analytical follow-up at the end of the postpartum period, Student’s *t*-test, the chi-square test, or the Fisher’s test, as appropriate, was used.

The Hb values were grouped into 10 sections to describe prescription and [8] to describe consumption.

Spearman’s rho was used to assess whether the prescribed oral iron regimen (daily dose, maintenance days, and the amount of total iron prescribed) corresponded to the Hb values seen at the beginning of postpartum. The variability of the prescription pattern is expressed by the width of the range and the scatter plot.

The Z-test with a difference of two proportions was calculated to measure the difference between the percentage of women with an iron prescription and women with iron consumption. Additionally, the Mann–Whitney U test was used to determine the adequacy of the sample.

The association between postpartum iron intake with iron deficiency, anemia, and maintenance of breastfeeding was estimated using the odds ratio and its 95% confidence interval, and its association with the level of fatigue and self-perceived maternal health status was measured with the Mann–Whitney U test.

## 3. Results

Information on Hb and iron prescription was collected at the beginning of postpartum from 1066 women. In 1019 of them, postpartum iron consumption was documented and in 966 (90.6%), information was obtained on analytical and clinical data at the end of the postpartum period.

The sociodemographic characteristics of the women with the analytical control at the beginning and end of postpartum are presented in Table 1. The mean age of the 1066 women in which postpartum follow-up began was 33.4 years (77.2%). They were Spanish and 50.7% were primiparous. Vaginal and spontaneous delivery occurred in 65.9% of the subjects. During pregnancy, 1.7% had anemia in the first trimester, 11.4% in the second, and 12.0% in the third. The prevalence of anemia at the beginning of postpartum was 24.5% (CI 21.8–27.1%). Iron was prescribed to 83.1% of the women during pregnancy and to 81.2% postpartum (Table 1).

There were no significant differences in the parity, type of delivery, existence of iron deficiency or anemia in pregnancy, and iron intake in pregnancy among the women repeating (or not) the laboratory test six weeks postpartum. The women with laboratory tests at six weeks were older and more often Spanish (*p* < 0.01). There were also no significant differences in the prevalence of anemia (Hb < 10) at the beginning of postpartum, but the percentage of women with an iron prescription was lower among the women who did not repeat the blood test (Table 1).

### 3.1. Iron Prescription

There were 51 women (4.8%) with Hb values < 8, 261 (24.5%) with Hb < 10, 547 (50%) with Hb < 11, and 855 (80.2%) with Hb < 12 24 h after delivery.

Oral iron was prescribed to 81.2% of women after delivery, 98.1% of women with anemia, and 75.8% of women without anemia. With regard to the prescribed preparations, 63.8% were composed of ferrous iron, and 36.2% of ferric iron. Before hospital discharge, 46 women with anemia (17.6%) received IV iron and 25 (9.6%) received a packed red blood cell transfusion, all of whom were also prescribed oral iron postpartum.

The higher the Hb figures, the greater the number of women without a prescription for oral iron, and this association is statistically significant (*p* < 0.01). Up to an Hb value of 10.9, the percentage of women without a prescription did not exceed 2.8%, in the range between 11 and 11.9, it reached 11.4%, and from Hb values ≥ 12, it was higher than 71% (Table 2).

Regarding the prescribed regimen, the initial Hb value weakly correlates with the daily iron dose (Spearman’s rho = −0.23, *p* < 0.01), and it is moderately correlated with the duration of treatment and the total iron prescribed (Spearman’s rho = −0.58, *p* < 0.01).

**Figure 1:**
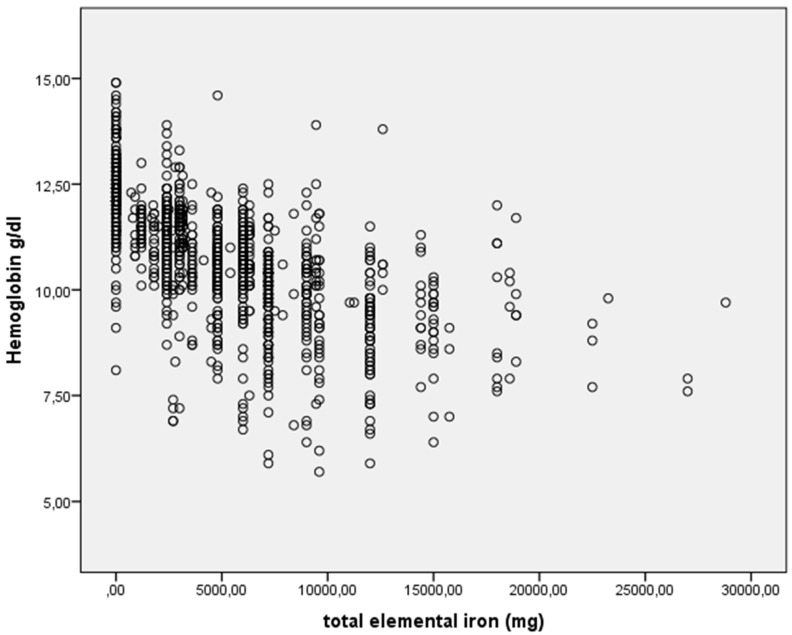
Amount of total elemental iron prescribed postpartum in each hemoglobin value. Hemoglobin g/dL = Initial Hb measured in the first 24 h postpartum. Total elemental iron (mg) = a daily dose of elemental iron in mg. was multiplied by treatment time in days (minimum = 0 mg and maximum = 28.800 mg).

The ranges of the prescription guidelines (dose, duration, and total iron) are wide in all of the Hb ranges (Table 2). In the three most frequent Hb cut-off points (Hb = 10.4 in 39 women, Hb = 11.4 in 35 women, and Hb = 9.4 in 20 women), we found that, with Hb = 10.4, the daily dose varied from 30 to 310 mg of elemental iron, the duration from 30 to 90 days, and the total amount of iron from 1800 to 18,600 mg; that is, the maximum prescribed dose was 10 times higher than the minimum doses; if Hb = 11.4, then the variation was 0 (iron is not prescribed) at a maximum daily dose of 105 mg, a duration of 90 days, and a total amount of elemental iron of 9000 mg; and if Hb = 9.4, then the daily dose ranged from 30 to 180 mg of elemental iron, from 60 to 180 days of treatment, and from 2400 mg of total elemental iron to 18,900 mg, which is almost eight times higher (data not shown). The dispersion of the total prescribed oral iron values is shown in Figure 1.

### 3.2. Actual Consumption

The proportion of women who consumed iron (48.7% (CI 45.6–51.8)) was 1.7 times lower than the proportion of women with an iron prescription (81.4% (IC 78.9–83.8)). In the group of women with anemia, it was 1.1 times lower, and in the group without anemia, 2.1 times lower. Among the women who took iron, whether or not they had anemia, the daily dose, days, and total iron amount consumed were always significantly lower than the prescribed guideline (*p* < 0.01) Table 3.

### 3.3. Association between Iron Consumption and Maternal Health

At six weeks, the prevalence of anemia was 7.0% (CI 5.3–8.7), iron deficiency (SF < 15 µg/L) was 3.8% (CI 2.6–5.1), anemia and iron deficiency comprised 1.4% (CI 0.6–2.3). All the cases of anemia were mild: the lowest value was Hb = 10.2 and, in 85.3% of the anemics, the Hb ranged between 11 and 11.9.

Table 4 depicts the health results of mothers either consuming or abstaining from iron in the puerperal period.

Regarding women with anemia at the beginning of postpartum, those who did not take iron were more likely to reach the end of postpartum with SF < 15 µg/L (OR = 3.6 (CI 1.3–10.3), *p* = 0.02), anemia (OR = 3.0 (CI 1.3–7.2), *p* = 0.01), and abandoning breastfeeding (OR = 2.4 (CI = 1.0–5.9), *p* = 0.04) than women who took iron. No significant differences were observed in the values of the state of health and fatigue between them.

Regarding women without anemia at the beginning of postpartum, those who did not take iron had a greater probability of iron deficiency (OR = 1.2 (CI = 0.4–4.0), *p* = 0.76). However, they also had a lower probability of anemia (OR = 0.8 (CI 0.3–2.1), *p* = 0.66) and abandoning breastfeeding (OR = 0.7 (CI 0.4–0.9), *p* = 0.04) than women who took iron. No significant difference was found in the level of fatigue, but the women’s self-perceived health status was significantly worse among the women taking iron.

In the subgroup of women with Hb values = of 10–10.9 who did not take iron, 95% reached six weeks without iron deficiency and 91% without anemia, although they had a higher probability of iron deficiency (OR = 2.4 (CI 0.6–10.8), *p* = 0.22) and anemia (OR = 4 (CI 1.2–13.9), *p* = 0.01)) than women who took iron with the same Hb values. If the value was 11–11.9 and the women did not take iron, the percentage of women without iron deficiency increased from 95% to 98% and the percentage of women without anemia grew from 91% to 99.5% compared with the previous subgroup. In this second subgroup, the probability of iron deficiency continues to be higher in women who did not take iron (OR = 1.8 (CI 0.2–16.4), *p* = 0.22), but the probability of anemia was lower (OR = 0.2 (CI 0.0–2.4), *p* = 0.22). No differences observed in fatigue in the two subgroups, Hb = 10–10.9 and Hb = 11–11.9; however, in the latter, women who took iron reported a worse health status at six weeks than those who did not.

## 4. Discussion

In this research, we described the prescription and actual iron consumption postpartum, and its association with maternal health status. The iron prescription guidelines showed a weak or moderate association with the initial postpartum Hb value, and, for the same Hb values, it showed wide variability in the daily dose, maintenance time, and total iron. The percentage of women who took iron and the doses they took were much lower than those that have been established in the iron prescription guideline. We found that iron intake is associated with a lower likelihood of iron deficiency, anemia, and abandoning breastfeeding if mothers initiate the postpartum period with anemia. Iron intake is associated with a lower probability of iron deficiency and anemia if mothers do not have anemia, but with a greater probability of abandoning breastfeeding and a poorer perception of health.

We found remarkable variability in the prescription of iron. Measuring Hb in all women after delivery does not seem to avoid variability in the iron prescription criteria. Other studies also found high variability in the prescription of oral iron to women with anemia and/or iron deficiency due to the low adherence of professionals to current guidelines [14], or the lack of them [18]. It is likely that in addition to the lack of unanimous criteria, there is a trivialization of the harmful effects of excessive oral iron intake, such as intestinal inflammation, dysbiosis, and the potential growth of enteropathogens [19]. This would explain the high percentage of mothers who were prescribed iron, even with initial Hb levels that were above 12 g/dL.

Gastrointestinal side effects secondary to iron intake are common, and they are a major cause of treatment discontinuation [20]. Among them, constipation causes discomfort and decreases the quality of life, especially in mothers with perineal lesions, fissures, or hemorrhoids [21]. We found that almost half of the mothers who were prescribed iron decided not to take it, and when they did, they reduced the daily dose and the frequency of consumption. However, this low adherence was not accompanied by moderate or severe anemia at the end of postpartum. It only reached 7% with mild anemia and 3.8% with iron deficiency; both of these situations will likely decrease in the following weeks [22,23].

Our study corroborates that postpartum iron supplementation reduces the risk of iron deficiency and anemia [7,11,24,25,26]. However, in women with initial Hb < 10 g/dL, supplementation may be necessary to achieve Hb ≥ 12 levels, whereas in women with Hb ≥ 10, the process of blood hemoconcentration, the increase in iron reserve from erythrocyte mass, and postpartum iron savings, amenorrhea compensates for the small iron loss due to breastfeeding, and it may be sufficient for preventing anemia at six weeks [23,26]. In addition, the greater the iron deficiency, the greater its uptake at the intestinal level, mediated by the concentration of hepcidin; hence, the mothers that would benefit most substantially from taking iron would be those who need it the most [27,28].

In this study, iron supplementation did not reduce fatigue, regardless of the initial Hb value. It also did not improve the perception of health. Furthermore, in mothers without anemia at the beginning of postpartum consuming iron, we observed a worse perception of health than those who were not taking it. This effect was mainly observed in women with an Hb value between 11 and 11.9. Among them, those who feel better may stop taking iron, whereas those who feel worse trust that iron—which has been prescribed—will be able to help, even if anemia is not the cause of their discomfort. Alternatively, having sufficient iron status leaves more unabsorbed iron in the intestinal lumen, which can be potentially harmful [29,30]. These data corroborate the findings of other studies, where neither the initial Hb level nor iron supplementation have been shown to exert an impact on fatigue or the health and quality of life of mothers [31,32,33,34].

In this study, the abandonment of breastfeeding was higher among the group of mothers with anemia that did not take iron, as well as the group without anemia who did take it; in this second group, a worse perception of health was also found, which could be related. In 2006, Rioux et al. found an association between the abandonment of breastfeeding and lower Hb values, but also with a low economic and educational level of the parents [35]. In 2017, Horie et al. reported that Hb levels < 9 increase the failure to initiate exclusive breastfeeding [36]. In other studies, Hb elevation after blood transfusion did not improve breastfeeding continuity [37]. Breastfeeding is a complex behavior, and many factors interact in its abandonment; there is still insufficient evidence on the effect of maternal iron status on the continuity of breastfeeding [38].

Our results are consistent with the academic literature. Systematic reviews have confirmed the effect of consuming iron to improve hematological indices during pregnancy and postpartum, but the clinical significance of this improvement is unclear, and the association between iron status and poor outcomes for the woman and her child has not yet been described [26,39,40]. In poor African mothers with iron-deficient anemia at nine weeks postpartum, iron intake displayed a favorable effect on their mental health, cognitive function, and interaction with their child [3,4,5]. Another study in mothers with anemia and low socioeconomic status did not find an association between iron intake and fatigue or other symptoms of anemia at six weeks, but rather with perceived health status. In English mothers with anemia, they did not show more difficulties in interacting with their children at 4 weeks after delivery. Additionally, the relationship between maternal iron status and postpartum depression is unclear: some studies have shown an association [2] whereas others have not.

## 5. Conclusions

Our study provides information related to the wide variability in prescription practice and postpartum iron consumption. The effect of taking iron in women with and without anemia differs. In mothers with anemia, iron consumption reduces the risk of anemia, iron deficiency, and abandonment of breastfeeding six weeks after delivery, but it does not show a beneficial effect on fatigue or perceived maternal health status. In mothers without anemia, consumption increases the risk of abandoning breastfeeding, and it is associated with the worst state of health. In mothers without anemia, consumption increases the risk of abandoning breastfeeding, and it is associated with a poorer self-perceived health assessment by mothers.

## 6. Limits and Strength

This is the first study in Spain to observe the practice of iron prescription in the postpartum period and its possible impact, not only on biological markers (Hb and SF) but also on maternal symptoms (fatigue and perception of health). However, this study has important limitations—firstly, the thresholds adopted for the definitions of anemia and iron deficiency are disputed and there is no broad agreement on them. Furthermore, the sample studied not only includes healthy women but women with blood requests, postpartum infections, or hemoglobinopathies were also included. Finally, the health measure tools of the study: fatigue and self-perception of health are limited for the diagnosis of the level of maternal health, the use of other recommended tools in the postpartum period such as the Edinburgh Postnatal Depression Scale (EPDS) would have improved this assessment.

## Figures and Tables

**Table 1:** Sociodemographic characteristics of the cohort of women in the postpartum period (from the first to the sixth week postpartum).

Characteristics	Puerperal			
	First Visit (N = 1066)	Second Visit		
		With Blood Analysis * (N = 966)	Without Blood Analysis ** (N = 100)	*p*
Age (X, SD)	33.4 (5.3)	33.6 (5.2)	31.4 (6.5)	<0.01
Place of residence ***, (n, %)				
Urban Zone: High socioeconomic status	195 (18.3)	176 (18.2)	19 (19.0)	0.05
Urban Zone: Medium socioeconomic status	363 (34.1)	337 (34.9)	26 (26.0)	
Low level: Low socioeconomic status	250 (23.5)	237 (24.5)	21 (21.0)	
Rural zone	258 (24.2)	216 (22.4)	34 (34.0)	
Country (n, %)				
Spain	823 (77.2)	759 (78.6)	64 (64.0)	<0.01
Others	243 (22.8)	207 (21.4)	36 (36.0)	
Delivery (n, %)				
Primiparous	540 (50.7)	498 (51.6)	42 (42.0)	0.07
Multiparous	526 (49.3)	468 (48.4)	58 (58.0)	
Type of delivery (n, %)				
Eutocid delivery	702 (65.9)	634 (65.5)	68 (68.0)	0.95
Vacuum extraction	81 (7.6)	74 (7.7)	7 (7.0)	
Forceps	70 (6.6)	63 (6.5)	7 (7.0)	
Caesarean delivery	213 (20.0)	195 (20.2)	18 (18.0)	
Iron deficiency first trimester (n, %)				
No	972 (93.2)	886 (93.7)	86 (88.7)	0.06
Yes	71 (6.8)	60 (6.3)	11(11.3)	
First trimester anemia (n, %)				
No	1028 (98.3)	934 (98.5)	94 (95.9)	0.06
Yes	18 (1.7)	14 (1.5)	4 (4.1)	
Iron deficiency second trimester (n, %)				
No	562 (53.6)	513 (53.9)	49 (50.5)	0.52
Yes	486 (46.4)	438 (46.1)	48 (49.5)	
Second trimester anemia (n, %)				
No	932 (88.6)	844 (88.4)	88 (90.7)	0.49
Yes	120 (11.4)	111 (11.6)	9 (9.3)	
Third trimester anemia (n, %)				
No	920 (88.0)	836 (88.1)	84 (86.6)	0.67
Yes	126 (12.0)	113 (11.9)	13 (13.4)	
Anemia at 24 h postpartum (n, %)				
No	805 (75.5)	724 (74.9)	81 (81.0)	0.180
Yes	261 (24.5)	242 (25.1)	19 (19.0)	
Prescription of iron in pregnancy (n, %)				
No	180 (16.9)	159 (16.5)	21 (21.0)	0.25
Yes	886 (83.1)	807 (83.5)	79 (79.0)	
Postpartum iron prescription (n, %)				
No	200 (18.8)	160 (16.6)	40 (40.0)	<0.01
Yes	866 (81.2)	806 (83.4)	60 (60.0)	

* Blood test includes complete blood count and serum ferritin value recorded in the woman’s electronic health record. ** Of the 100 women without a blood test, 27 did not attend the consultation at six weeks and 73 attended the consultation, but without having previously done the blood test. *** Socio-economic level of the urban place of residence is established according to the appraised value of the home in euros per square meter (high > 2500 €/m^2^); medium (2500–1000 €/m^2^) and low (<1000 €/m^2^).

**Table 2:** Treatment for anemia in the postpartum period: women treated, types of treatment and prescribed oral iron guidelines.

Initial Hb (g/dL)	Women n (%)	Treatment Prescription: n (%)					Oral Iron Regimen (mg of Elemental Iron)					
		Without Treatment	With Prescribed Treatment				Dose/Day (mg)		Duration (Days)		Total Fe (mg)	
			T + IV + O	T + O	IV + O	O	P50	Range	P50	Range	P50	Range
<6	3 (0.3)			3 (100)			160	120	60	30	9600	4800
6–6.9	14 (1.3)		4 (28.6)	8 (57.1)	1 (7.1)	1 (7.1)	100	170	82.5	45	9000	12,300
7–7.9	34 (3.2)		1 (2.9)	8 (23.6)	19 (55.9)	6 (17.6)	160	280	75	60	9600	24,300
8–8.9	69 (6.5)	1 (1.4)		1 (1.4)	19 (27.5)	48 (69.6)	130	260	75	152	9000	20,100
9–9.9	141 (13.2)	4 (2.8)			2 (1.4)	135 (95.7)	100	280	75	150	9000	26,400
<10	261 (24,5)	5 (1.9)	5 (1.9)	20 (7.6)	41 (15.7)	190 (72.8)	100	280	75	152	9000	26,400
10–10.9	286 (26.8)	5 (1.7)				281 (98.3)	80	280	60	150	6000	177,000
11–11.9	308 (28.9)	35 (11.4)				273 (88.6)	80	270	30	164	3000	18,100
12–12.9	161 (15.1)	115 (71.4)				46 (28.6)	80	270	30	72	3000	17,280
13–13.9	39 (3.7)	30 (76.9)				9 (23.1)	80	65	30	90	2400	11,400
14–14.9	11 (1.1)	10 (90.9)				1 (9.1)	80	–	60	–	4800	–
≥10	805 (75.5)	195 (24.2)				610 (75.8)	80	280	30	180	3000	18,180

Initial Hb measured in the first 24 h postpartum (minimum Hb value = 5.7 and maximum value = 14.9). Hemoglobin values are broken down into 10 sections: 5 correspond to the diagnosis of anemia (Hb <10 g/dL) and 5 to without anemia (Hb ≥ 10 g/dL). Types of treatment: T = transfusion, IV = intravenous iron, O = oral iron. P50 = 50th percentile (median) and range = interquartile range.

**Table 3:** Description of the prescribed regimen and the actual iron consumption of 1019 postpartum women (6 weeks).

Initial Hb (g/dL)	Prescription (*p*) vs. Consumption (C)				Dose/Day			Days			Fe Total		
	N	n	% (95% CI)	*p* ^#^	P50	IQR	*p* ^##^	P50	Range	*p* ^##^	P50	Range	*p* ^##^
<8	51	P = 51	100	0.13	160	280	0.01	75	75	<0.01	9600	24,300	<0.01
		C = 47	92.2 (81.1–97.8)		80	270		34	31		2960	9480	
8–8.9	64	P = 63	98.4 (91.6–100)	0.07	160	260	0.06	75	152	<0.01	9000	20,100	<0.01
		C = 57	89.1 (80.6–97.5)		80	180		33	33		2640	7000	
9–9.9	133	P = 129	97.0 (92.5–99.2)	0.03	100	280	0.08	75	150	<0.01	9000	26,400	<0.01
		C = 119	89.5 (83.9–95.1)		80	280		34	29		2900	9170	
<10	248	P = 243	98.0 (95.4–99.3)	<0.01	100	280	<0.01	75	152	<0.01	9000	26,400	<0.01
		C = 223	89.9 (86.0–93.9)		80	280		34	34		2800	9760	
10–10.9	273	P = 268	98.2 (95.8–99.4)	<0.01	80	280	0.03	60	150	<0.01	6000	17,700	<0.01
		C = 172	63.0 (57.1–68.9)		80	173		30	38		2050	5910	
11–11.9	299	P = 265	88.6 (84.9–92.4)	<0.01	80	270	0.09	60	60	<0.01	3150	17,100	<0.01
		C = 91	30.4 (25.1–35.8)		80	75		30	38		1800	3580	
≥12	199	P = 53	26.6 (20.2–33.0)	<0.01	100	220	<0.01	30	30	<0.01	3000	15,600	<0.01
		C = 10	5.01 (1.7–8.3)		100	25		30	7		3000	1380	
≥10	771	P = 586	76.0 (72.9–79.1)	<0.01	80	280	<0.01	60	150	<0.01	4800	17,700	<0.01
		C = 273	35.4 (32.0–38.8)		80	173		30	38		2080	5910	

Initial Hb measured in the first 24 h postpartum. Hemoglobin values were broken down into eight sections: four correspond to the diagnosis of anemia (Hb <10 g/dL) and four to those without anemia (Hb ≥ 10 g/dL). P50 = 50th percentile (median) and range = interquartile range ^#^ *p*-value of the difference in proportions; ^##^ *p*-value Mann–Whitney U test.

**Table 4:** Association between iron consumption in the first six weeks of gestation and iron deficiency, anemia, fatigue, and health status perceived by women in the cohort of 966 women with follow-up and laboratory tests at six weeks of childbirth.

Initial Hb	Iron Intake	Iron Deficiency (SF < 15 µg/L)			Anemia (Hb < 12 g/dL)			Breastfeeding			Fatigue		Health Condition	
		NOT n (%)	YES n (%)	OR (CI)	NOT n (%)	YES n (%)	OR (CI)	NOT n (%)	YES n (%)	OR (CI)	P50 (Range)	*p* ^#^	P50 (Range)	*p* ^#^
<10	NO	17 (73.9)	6 (26.1)	3.6 (1.3–10.3) *	12 (52.2)	11 (47.8)	3.0 (1.3–7.2) *	10 (43.5)	13 (56.5)	2.4 (1.0–5.9) *	19 (20)	0.34	75 (50)	0.48
	YES	195 (91.1)	19 (8.9)		177 (76.6)	54 (23.4)		52 (24.1)	164 (75.9)		19 (30)		75 (60)	
10–10.9	NO	94 (95.9)	4 (4.1)	2.4 (0.6–10.8)	91 (91.0)	9 (9.0)	4.2 (1.2–13.9) *	13 (13.0)	87 (87.0)	0.7 (0.4–1.4)	18 (21)	0.43	80 (40)	0.70
	YES	168 (98.2)	3 (1.8)		168 (97.8)	4 (2.2)		30 (17.4)	142 (82.6)		18 (21)		78 (40)	
11–11.9	NO	195 (98.0)	4 (2.0)	1.8 (0.2–16.4)	199 (99.5)	1 (0.5)	0.2 (0.0–2.4)	22 (11.2)	175 (88.8)	0.7 (0.4–1.4)	18 (31)	0.45	80 (45)	0.01 **
	YES	88 (98.9)	1 (1.1)		88 (97.8)	2 (2.2)		22 (25.6)	64 (74.4)		18 (24)		75 (40)	
≥12	NO	153 (100)	0 (0.0)	–	153 (99.4)	1 (0.6)	–	27 (17.0)	124 (83.0)	–	18 (24)	0.65	80 (46)	0.44
	YES	9 (100)	0 (0.0)		9 (100)	0 (0.0)		0 (0.0)	8 (100)		18 (3)		82.5 (35)	
≥10	NO	442 (98.2)	8 (1.8)	1.2 (0.4–4.0)	443 (97.6)	11 (2.4)	0.8 (0.3–2.1)	62 (13.8)	386 (86.2)	0.7 (0.4–0.9) *	18 (31)	0.42	80 (46)	0.02
	YES	265 (98.5)	4 (1.5)		263 (97.8)	6 (2.2)		52 (19.6)	214 (80.4)		18 (24)		78 (40)	

Initial Hb measured in the first 24 h postpartum. Hemoglobin values are broken down into eight sections: four correspond to the diagnosis of anemia (Hb <10 g/dL) and four to those without anemia (Hb ≥ 10 g/dL). P50 = 50th percentile (median) and range = interquartile range * *p*-value <0.05; ** *p*-value <0.01: ^#^ Mann–Whitney U test *p*-value.

## Data Availability

The data is available by contacting the corresponding authors.
